# Antiproliferative activity and induction of apoptotic by ethanolic extract of Alpinia galanga rhizhome in human breast carcinoma cell line

**DOI:** 10.1186/1472-6882-14-192

**Published:** 2014-06-17

**Authors:** Saeed Samarghandian, Mousa-Al-Reza Hadjzadeh, Jalil Tavakkol Afshari, Mohadeseh Hosseini

**Affiliations:** 1 Department of Basic Medical Sciences, Neyshabur University of Medical Sciences, Neyshabur, Iran; 2 Department of Physiology, Faculty of Medicine, Mashhad University of Medical Sciences, Mashhad, Iran; 3 Immunogentics and Cell Culture Department, Immunology Research Center, Bu-Ali Research Institute, Mashhad University of Medical Sciences, Mashhad, Iran; 4 Department of Biology, Payame Nour University of Tehran, Tehran, Iran

**Keywords:** Alpinia galanga L, Cytotoxicity, MCF-7, MRC-5, MTT

## Abstract

**Background:**

We investigated the potential of galangal rhizomes to induce cytotoxic and apoptotic effects in the cultured human breast carcinoma cell line, (MCF-7) in compare with the non-malignant (MRC-5) cells.

**Methods:**

Both cells were cultured in DMEM medium and treated with galangal rhizomes for three consecutive days. The percentage of apoptotic cells was determined by flow cytometry using Annexin-V fluorescein isothiocyanate.

**Results:**

The results showed that the ethanolic extract of galangal rhizomes decreased cell viability in the malignant cells as a concentration- and time- dependent manner. The IC_50_ values against MCF-7 were determined at 400.0 ± 11.7 and 170.0 ± 5.9 μg/ml after 48 and 72 h respectively. The morphology of MCF-7 cells treated with the ethanolic extract confirmed the cell proliferation assay results. Alpinia galanga induced apoptosis in MCF-7 cells, as determined by flow cytometry.

**Conclusions:**

We concluded that the extract of Alpinia galanga exerts pro-apoptotic effects in a breast cancer-derived cell line and could be considered as a potential chemotherapeutic agent in breast cancer.

## Background

Breast cancer is the second leading cause of cancer deaths among woman. Unfortunately, the development of resistance to chemotherapeutic agents is a common obstacle in the treatment of different types of cancers including breast cancer [1]. Several important drugs with different structures and mechanisms of anti-tumor activities fail to be effective due to the drug resistance [2] and also the failure of the conventional chemotherapy to affect a major reduction in mortality indicates that the new approaches are critically needed [3]. It has been recognized that a large groups of therapeutic agents can stop cancer cells proliferation by inducing apoptosis. The induction of apoptosis has been emphasized in anticancer strategies [4]. Apoptosis is a gene regulated phenomenon which is induced by many chemotherapeutic agents in cancer treatment [5]. It is characterized by a series of typical morphological features, such as nuclear and cellular convolution, chromatin condensation and the final disintegration of the cell into membrane-bound apoptotic bodies which are phagocytosed by neighboring cells [6]. Most normal cells can die by apoptosis but tumor cells very often have some defects in the apoptotic pathway, leading not only to the increase of tumor mass but also to tumor resistance to chemotherapy. Since chemotherapy and irradiation act primarily by inducing apoptosis, defects in the apoptotic pathway make the therapy less efficient [7]. Increasing evidences suggest that the related processes of neoplastic transformation involve alteration of the normal apoptotic pathway [8]. The major focus of the research in chemotherapy for cancer in recent times is the use of naturally occurring compounds with the chemopreventive and chemotherapeutic properties in the treatment of cancers [9,10]. Epidemiological studies suggest that a diet rich in antioxidants may help to prevent the development of breast carcinoma [11]. Excess generation of oxygen free radicals can cause oxidative damage to bimolecular resulting in lipid peroxidation, mutagenesis and carcinogenesis. All cells are exposed to oxidative stress, and thus oxidation and free radicals may be important in carcinogenesis at multiple tumor sites [12]. The antioxidant activity may be the result of the specific scavenging of reactive free radicals, scavenging of oxygen containing compounds such as hydrogen peroxide and chelating metals [13,14]. Phytochemical and dietary antioxidants may decrease the risk of much chronic disease such as cancer. Antioxidants scavenge free radicals, and consequently are a very special group of nutrition supplements [15]. Plants have played an important role as a source of effective anticancer agents, and it is significant that 60% of currently used anticancer agents are derived from natural sources including plants, marine organisms, and microorganisms [16]. Alpina galanga (galangal) is a well-known plant in the Southeast Asia. The rhizomes of Zingiberaceae family are widely used in many ancient countries in traditional medicine which is found to be effective in the treatment of diseases [17]. Their function have been broadly discussed and accepted in many traditional recipes. Alpinia galangal has bben studied by various researchers and a number of active constituents from the plant have been isolated and reported. Phenolic compounds such as flavonoids and phenolic acids are found abundantly in this plant [18]. The dominant components isolated from the rhizomes were galangoisoflavonoid [19], β-sitosterol diglucosyl caprate [20], methyleugenol, p-coumaryl diacetate, 1′-acetoxyeugenol acetate, trans-p-acetoxycinnamyl alcohol, trans-3,4-dimethoxycinnamyl alcohol, p-hydroxybenzaldehyde, p-hydroxycinnamaldehyde, trans-p-coumaryl alcohol, galangin, trans-p-coumaric acid, and galanganol B [21]. The major phytoconstituents which have been isolated from the rhizomes are acetoxychavicol acetate (ACA) and hydroxychavicol acetate (HCA) [22]. Rhizomes are lowest in fat but richest in carbohydrate [23]. The chemical investigation of A. galanga has led to the isolation of β-caryophyllene (17.95%) and β-selinene (10.56%), terpinen-4-ol [24], 4-allylphenyl acetate and β-bisabolene, 5-hydroxymethyl furfural (59.9%), benzyl alcohol (57.6%), methylcinnamate (9.4%), 3-phenyl-2-butanone (8.5%) and 1,2-benzenedicarboxylic acid (8.9%) [25]. A new phenylpropanoid, 4,4′[(2E, 2′E)-bis(prop-2-ene)-1,1′-oxy]-diphenyl-7,7′-diacetata [26], as well as p-coumaryl alcohol-γ-O-methyl ether (CAME) and p-coumaryl diacetate (CDA), has also been isolated from the plant [27,28]. Volatile oil of plant contained zerumbone (44.9%), β-farnesene, myrcene and 1,8-cineole, respectively [29,30]. Bicyclo (4.2.0) oct-1-ene, 7-exoethenyl (58.46%), trans-caryophyllene (7.05%), α-pinene (14.94%) with camphene (2.15%), germacrene (1.78%) and citronellyl acetate (1.41%) were reported in A.galanga as major components [31]. Several authors have studied the anti-inflammatory and analgesic effects of A. galanga in a variety of rheumatological conditions [26,27,32,33]. The extracts of A. galanga showed acetylcholinesterase-inhibitory [34], platelet-activating factor (PAF)-inhibitory [35], antimicrobial [36], antibacterial [37], anti-amoebic [38], antifungal [39], antioxidant [28] and apoptosis [40] activities. It has been reported that Alpinia plants possess antioxidant [41], anti-inflammatory [42], immunostimulating [43], antinociceptive [44], hepatoprotective [45] and anticancer [46] activities. However our information about anticancer activates of galangal is very few. To prevent initiation and promotion carcinogenesis, administration of nationally accruing agent is being increasingly appreciated. In this study, the anticancer effects of the ethanolic extract of galangal on the cultured human breast carcinoma, MCF-7 cells, was investigated.

## Methods

### Chemicals and reagents

3-(4, 5-Dimethylthiazol-2-yl)-2, 5-diphenyltetrazolium bromide (MTT) was purchased from Amerso (USA). Dulbecco’s modified Eagle’s medium (DMEM) was purchased from Gibco BRL (Grand Island, NY, USA). Annexin V/fluorescein isothiocyanate (FITC) was obtained from Invitrogen Corporation (Camarillo, CA, USA). Fetal bovine serum was purchased from PAA Laboratories GmbH, Austria.

### Preparation of the galangal extract

Protocols of current study were approved by the Ethical Committee of Mashhad Medical University. The Fresh rhizomes of alpinia galanga used in this study was collected from a private garden in the flowering period in india and identified by botanists in the herbarium of Ferdowsi University of Mashhad and also has been deposited in the herbarium. Voucher specimen is deposited in the specially maintained herbarium, Department of Botany, Ferdowsi University of Mashhad. The cleaned fresh rhizomes were cut into small pieces and air-dried. The dried powder (50 g) was mixed with ethanol (97%) in a balloon; the balloon was shaken for 3 days at room temperature. The preparation was then filtered off through a Gauze mesh and the solvent was dried by evaporation under reduced pressure at 45°C. The final product yielded 12% w/w dried extract; it was stored in a refrigerator until the experiment.

### Cell culture

The human breast adenocarcinoma cell line (MCF-7) and the human fetal lung fibroblast cell line (MRC-5) were obtained from Pasteur Institute (Tehran, Iran). The cells were grown either in 96-well tissue (TC) plate (NUNC, Wiesbaden, Germany) or in 25-cm^2^ TC flasks (NUNC, Wiesbaden, Germany), cells were cultured in CO_2_ incubator MCO-17AI (Sanyo Electric Co., Ltd, Japan) at 37°C in 95% humidified atmosphere enriched by 5% CO_2_ and subcultured every 3 to 4 days. The malignant (MCF-7) and nonmalignant cells (MRC-5) were cultured in DMEM supplemented with 10% (v/v) fetal bovine serum (Gibco–Invitrogen), 100 U/ml of penicillin (Gibco–Invitrogen), and 100 μg/ml streptomycin (Gibco–Invitrogen).

### Cell viability assay

Cell viability was measured using the MTT assay, which is based on the conversion of MTT to formazan crystals by mitochondrial dehydrogenises [6]. Briefly, both cell lines were plated at a density of (1 × 10^3^ cells/ml) in 96-well plates. Cells were seeded overnight, and then incubated with various concentrations of galangal rhizomes extract (0, 125, 250, 500, and 750 μg/ml) for 24, 48 h and 72 h. For MTT assay, after treatment with the galangal extract for 24 and 48 and 72 h, 10 μl MTT was added into each well. After removing the medium, the cells were then labeled with MTT solution) 5 mg/ml in PBS) for 4 h and the resulting Formosan was solubilized in 100 μl dimethyl sulfoxide (DMSO). Absorbance was measured at 570 nm using an automated microplate reader (Bio-Rad 550). The absorption was measured at 570 nm (620 nm as a reference) in an ELISA reader. The cytotoxic effects of the galangal extract on the cell lines (MCF-7 and MRC-5) were expressed as the IC_50_ value (the drug concentration reducing the absorbance of treated cells by 50% with respect to untreated cells). All experiments were carried out in triplicate

### Morphological studies of cell lines using the normal inverted microscope

Morphological studies using the normal inverted microscope were carried out to observe the morphological changes of cell death in MCF-7 and MRC-5 cell lines elicited by the ethanolic extract of galangal. Different concentrations of galangal extract (0, 125, 250, 500, and 750 μg/ml) for 24, 48 h and 72 h were used for the morphological studies. The untreated cells served as the negative control. The morphological changes of the cells were observed under the normal inverted microscope after 24 and 48 h post-treatment.

### Annexin V/PI staining and flow cytometry analysis

Apoptotic cell death was measured using a flouresin isothiocynate (FITC)-conjugated Annexin V/PI assay kit by flow cytometry. Briefly, MCF-7 cells (1 × 10^6^ cells) treated with the galangal extract at concentrations of 250 and 500 μg/ml for 48 h were harvested and washed twice with cold PBS (phosphate-buffered saline) resuspended in 100 μl binding buffer, and stained with 5 μl of FITC- conjugated Annexin V (10 mg/ml) and 10 μl of propidium iodide (PI) (50 mg/ml). The cells were incubated for 15 min at room temperature in the dark, 400 μl of binding buffer was added, and the cells were analyzed (FACScan, Becton- Dickinson, USA). The MCF-7 cells were gated separately according to their granularity and size on forward scatter (FSC) versus Side Scatter (SSC) plots. The early and late apoptosis were evaluated on fluorescence 2 (FL2 for PI) versus fluorescence 1 (FL1 for Annexin) plots. Cells stained with only annexin V were evaluated as being in the early apoptosis; cells stained with both annexin V and PI were evaluated as being in the late apoptosis or in the necrotic stage [7,47].

### Statistical analysis

All results were expressed as mean ± SEM. The significance of difference was evaluated with ANOVA and Bonfrroni’s test. A probability level of P < 0.05 was considered statistically significant.

## Results

### Morphological evaluation

**Figure 1:**
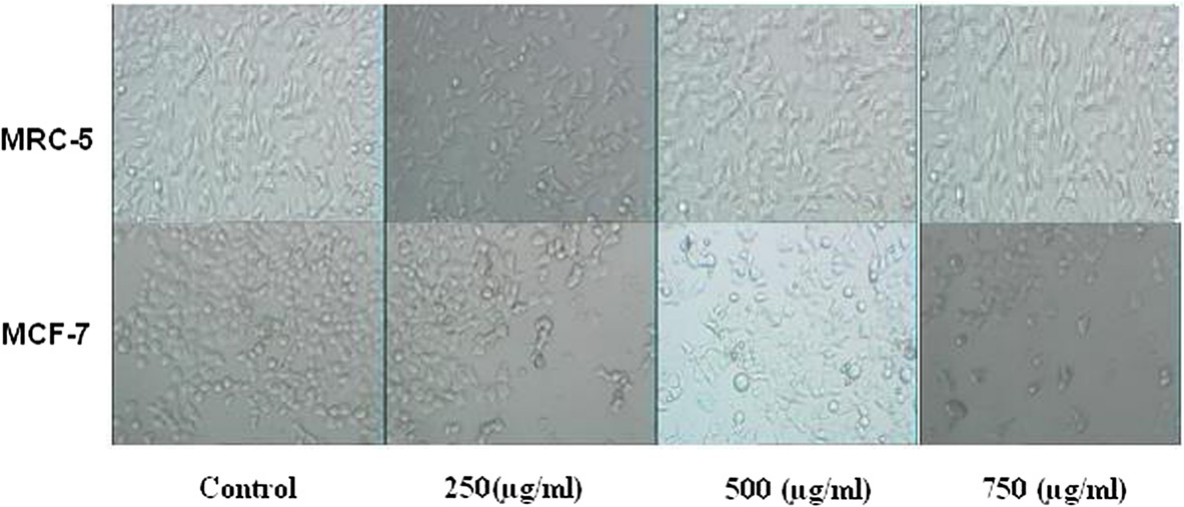
Effect of galangal extract on morphological evaluation in MCF-7 and MRC-5 cells after 48 h treatment.

**Figure 2:**
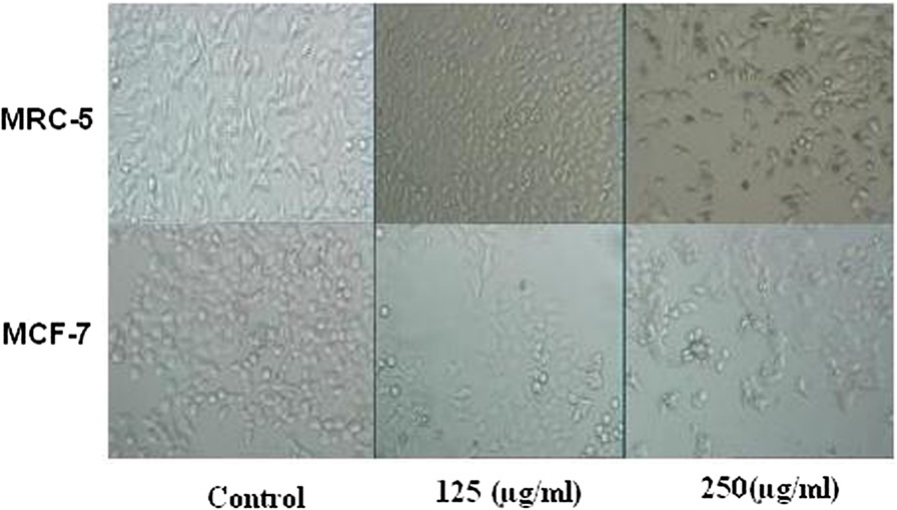
Effect of galangal extract on morphological evaluation in MCF-7 and MRC-5 cells after 72 h treatment.

To discriminate between the early and late effects of galangal rhizomes action, the malignant (MCF7) and non-malignant control (MRC-5) cells were exposed to increasing the galangal rhizomes concentrations for 24, 48 and 72 h (Figure 1).After 48 h of incubation with the ethanolic extract of galangal rhizomes (250, 500, and 750 μg/ml) morphologic changes were observed in MCF-7 cells versus MRC-5 cells which is consisting of reduction in the number of living cells, volume and rounding until the nucleus constituted the majority of the cellular volume. No morphological changes were detected in MRC-5 cells. Our data showed the reduction of MCF-7 cells compared with MRC-5 cells. Our MTT result showed that this cytotoxicity was increased at higher concentrations (Figure 1), so that, the galangal rhizomes treated MCF-7 (125 μg/ml) were damaged but there were no morphological changes in galangal rhizomes treated MRC-5 cells at the same concentration, this effect again became obvious at the other concentration (250 μg/ml). Although the morphological features were not dramatically changed in the MCF-7 versus MRC-5 cells after 24 h at lower concentration(data was not shown), however after 72 h, at concentration (125, 250 μg/ml), there was observationally sever difference between galangal rhizomes - treated MCF-7 and MRC-5 cells at morphological level (Figure 2).

### Effects of galangal on cell viability

**Figure 3:**
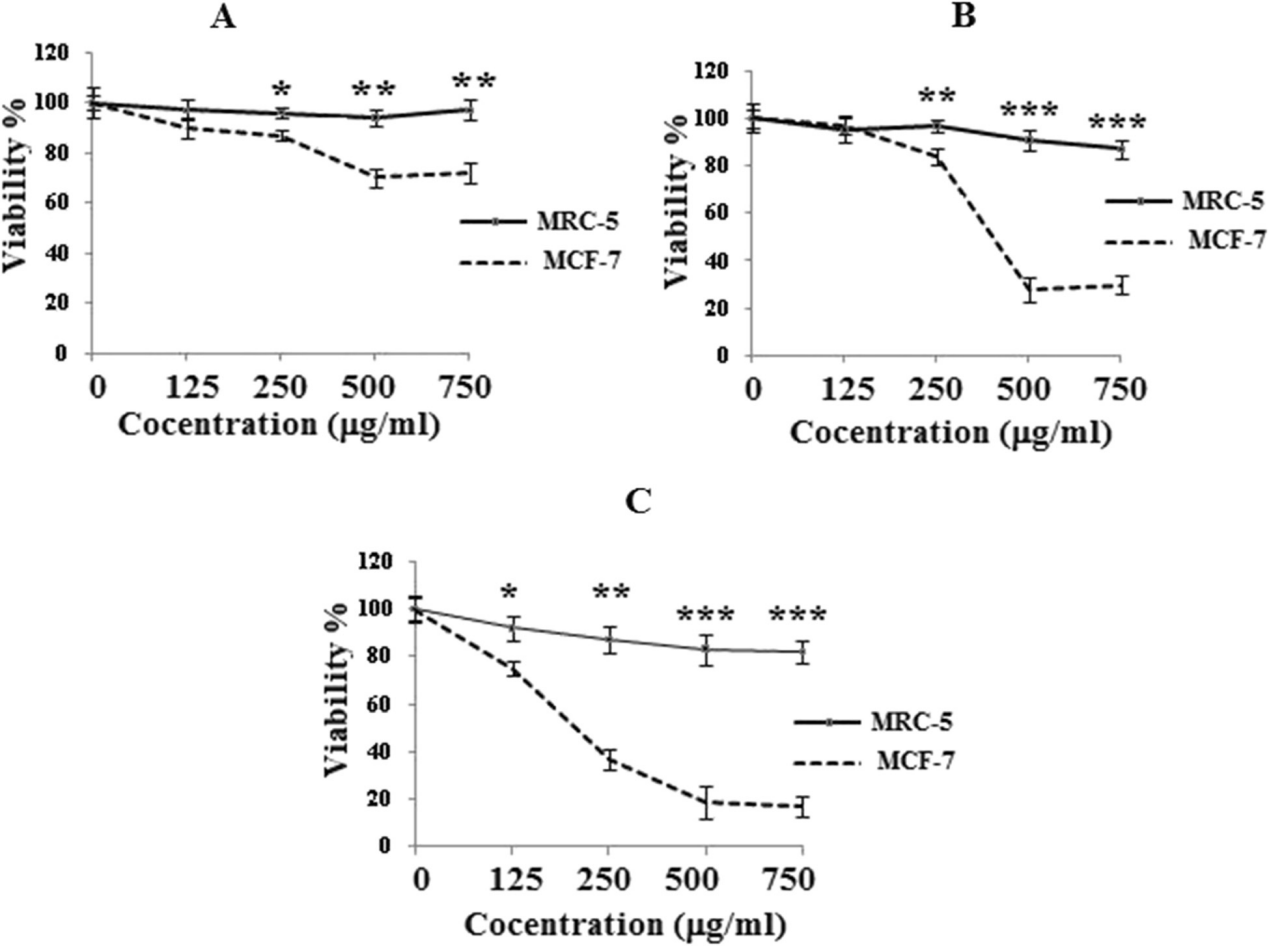
**Comparison cytotoxic effect of galangal extract on cell viability of MCF-7 and MRC-5 cells.** Cells were treated with different concentrations of the extract for 24 h **(A)**, 48 h **(B)** and 72 h **(C)**, results are mean ± SEM(n = 6). *p < 0.05, **p < 0.01 and ***p < 0.001 compared to control (MRC-5).

In order to evaluate the effect of the ethanolic extract of galangal rhizomes on the growth of MRC-5 and MCF-7 human breast cancer cells, the cells were treated with different concentrations of galangal rhizomes for 3 consecutive days and their growth inhibitory effects were compared. The impact of the galangal rhizomes extract on cell viability was quantitated by the MTT assay. The ethanolic galangal rhizomes extract showed significantly a high growth inhibitory effects on MCF-7 cell line in dose-dependent manner (p < 0.001) compared with MRC-5 cells. The ethanolic extract (250, 500, 750 μg/ml) decreased the cell viability in the malignant cells but not in non-malignant cells after 24 h (p < 0.01) (Figure 3A). This toxicity was consistent with the morphologic changes. However, the extract at the different concentrations (125 μg/ml) could not decrease the cell viability in compare with the MRC-5 cells. After 48 h, the higher concentrations of ethanolic extract (500, 750 μg/ml) dramatically decreased the cell viability in the MCF-7 cells (p < 0.001) (Figure 3B). After 72 h, again the ethanolic extract statistically deceased cell viability in MCF-7 cells versus MRC-5 cells even at the lower concentration (125 μg/ml) (Figure 3C). The dose inducing 50% cell growth inhibition (IC_50_) against MCF-7 cells was determined as 400.0 ± 11.7 and 170.0 ± 5.9 μg/ml after 28 and 48 h respectively (Table 1).

**Table 1:** **Doses induce 50% cell growth inhibition (IC**_**50**_**) of ethanolic galangal extract against MCF-7 cell line**

IC50	48 h	72 h
MCF7	500 (μg/ml)	250 (μg/ml)

### Induction of apoptosis in MCF-7 cells by galangal

**Figure 4:**
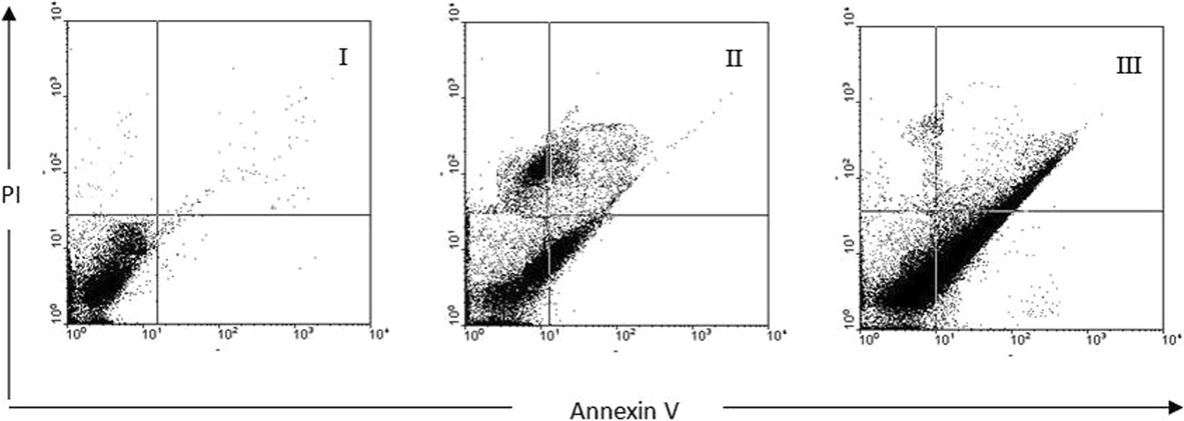
**Assessment of apoptosis by Annexin V/PI staining on the human breast adenocarcinoma cell line (MCF-7).** The cells were treated with 250 and 500 μg/ml galangal extract for 48 h (symbol II, III) or media (control symbol I), and apoptosis was examined with flow-cytometry after Annexin V-PI double staining. The necrotic cells lost cell membrane integrity that permits PI entry .Viable cells exhibit Annexin V (-)/PI (-); early apoptotic cells exhibit Annexin (+)/PI (-); late apoptotic cells or necrotic cells exhibit Annexin V (+)/PI (+).

**Figure 5:**
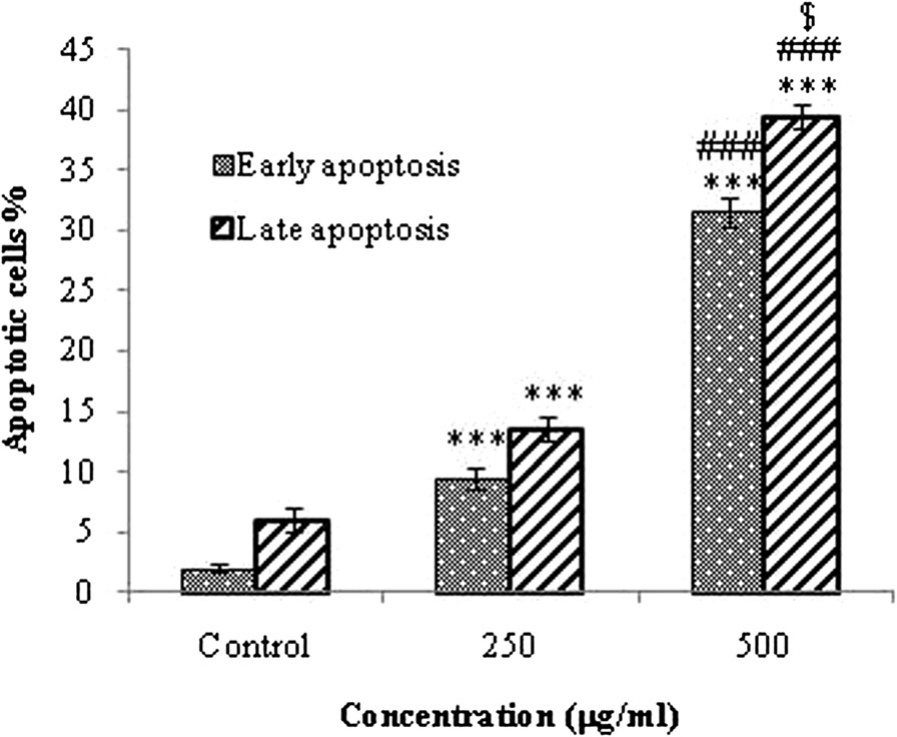
**Assessment of apoptosis by Annexin V/PI on the human breast adenocarcinoma cell line (MCF-7) after 48 h.** Percentage of cell death based on the assessment of apoptosis by Annexin V/PI. ***, ### p < 0.001 compared with control and the other dose respectively. $ p < 0.05 compared with early apoptosis.

In order to assess the rate of cell death by apoptosis or necrosis, the galangal-treated MCF-7 cells were assayed by Annexin V-FITC/PI dual staining (Figure 4). As it can be seen in Figure 4, the malignant cells were treated with concentrations of 250 and 500 μg/ml galangal for 48 h, then the cells were harvested, and apoptosis was examined by flow cytometry. The cells were treated with 250 and 500 μg/ml galangal for 48 h(symbol П, ПI) or media (control symbol I), and apoptosis was examined with flow-cytometry after Annexin V-PI double staining. The necrotic cells lost cell membrane integrity that permits PI entry. The viable cells exhibit Annexin V (-)/PI (-); early apoptotic cells exhibit Annexin (+)/ PI (-); late apoptotic cells or necrotic cells exhibit Annexin V (+)/PI (+). To study roles of galangal in apoptosis, the ethanolic extract of galangal was used to setup apoptosis system on the MCF-7 cells. Quantitative analysis using annexin V/PI assay further showed that the proportion of the early-stage apoptotic cells (annexin V+/PI-) increased significantly from 9.5% to 31.5%, while proportion of the late stage apoptotic cell (annexin V+/PI+) increased significantly from 13.5% to 39.5% in the cells were treated with 250 and 500 μg/ml galangal extract for 48 h respectively. Apoptosis induced by 250 and 500 μg/ml of galangal was statistically higher than control, and the percentage of early and late apoptotic cells significantly increased by increasing the galangal concentrations (p < 0.05). At 48 h, the total early apoptotic cells were significantly elevated from 2% (control) to 9.5 and 31.5%.at 250 and 500 μg/ml respectively. The percentage of the late apoptotic/necrotic cells increased from 6% (control) to 13.5 and 39.5% at 250 and 500 μg/ml respectively (Figure 5). Although no significant difference was detected between the percentage of early and late apoptotic cells at concentration of 250 μg/ml, however, the number of late apoptotic cells versus to early apoptotic cells at concentration of 500 μg/ml galangal treated cells was statically significant (p < 0.001); thus, the percentage of late apoptotic cells increased significantly versus the percentage of early apoptotic cells.

## Discussion

Several studies have shown that a number of herbal medicine or mixtures have anticancer potential in vitro and in vivo. They might be good candidate for the development of anti-cancer drugs [48]. Most chemo-preventive agents suppress the various stages of signaling events of carcinogen-induced transformation of normal cells, with striking inhibition of diverse cellular events related to cancer development. Inhibition of cancer cell proliferation, growth factor signaling, induction of apoptosis in cells with malignant potential, etc., contributes for chemo-preventive properties. Increasing evidences suggest that most chemo-preventive agents are capable of inducing apoptosis in the target cancer cells either with mitochondrial or by an uncharacterized mechanism in certain cases [49,50]. Search for novel agents from plants with promising features of apoptosis induction in cancer cells is an attractive strategy.

Alpinia galangal is known to possess flavanoids such as kaempferol, kaempferide, and galangin as well as the terpenoids 10-acetoxy chavicol acetate, 10-acetoxy eugenol, galanal A, galanal B, and galanolactone [47]. We are reporting for the first time that the ethanolic extract of galangal rhizome-induced cytotoxicity and apoptosis on MCF-7 cells versus MRC-5 cells. Much effort has been directed toward the effect of the galangal extract on apoptosis and understanding their mechanisms of action. To study whether the result of MTT assay was due to apoptosis, cells were stained with Annexin V-FITC and PI. The apoptosis evoked by the extract was confirmed by the annexin V–FITC (Figure 3). In the present study, the galangal-induced apoptosis was involved in cell death. Apoptosis is characterized by distinct morphological features including the chromatic condensation, cell and nuclear shrinkage, membrane blabbing, and oligonucleosomal DNA fragmentation [51]. As shown in Figures 3 and 4, the Alpina extract at 250 and 500 μg/ml induced significant cell toxicity in MCF-7 cells in a dose-dependent manner. Therefore, this anti-proliferative effect was due to the induction of apoptosis as shown by the annexin-V-flow cytometric approach. But, apoptosis only partially contributed in this toxicity, and it might be conducted that nonapoptotic cell death can also be involved in the galangal-induced toxicity in these cells. Although the significant of nonapoptotic cell death in chemotherapy remains largely unclear, it is believed that the nonapoptotic cell death is important under conditions in which apoptosis is inhibited [52,53]. Overall, this study showed that the alpina extarct may contain bioactive compounds that inhibit the proliferation of breast cancer cell lines (MCF-7) with the involvements of apoptosis or programmed cell death. Further studies are needed to fully recognize the mechanism involved in cell death, alpina extract could be considered as promising chemotherapeutic agent in lung cancer treatment.

The fresh rhizomes of galangal has been assessed for free radical scavenging activity against 1, 1- diphenyl-2-picrylhydrazyl (DPPH) radical and cytotoxic activity. It has been reported that Alpinia plants possess antioxidant [33]. In vitro cell proliferation inhibition test using MTT viability assay confirmed that the ethanolic extract at 24, 48 and 72 h, has strongly cytotoxic activity against MCF-7 cell line but not in nonmalignant cells (MRC-5) (Figure 3). The present results were consistent with previous studies indicating that MeOH and CH_2_Cl_2_ extractable of Alpinia possess significant cytotoxicity against CORL23 human large-cell carcinoma with IC_50_ values of 13.3 and 5.4 μg/ml respectively [48]. xPresent data were also consistent with previous studies indicating that galangal rhizomes and its ingredients possess cytotoxcity and free radical scavenging activities [54,55]. Phytochemical studies showed that of the many chemical constituents isolated from Alpinia, diaryheptanods, are among the characteristic compounds [56]. Curcumin, a well-known diarylheptanoid, has been postulated to be of potential use not only in cancer chemo-prevention but also in chemotherapy [57]. Two new diarylheptanoids 1, 2 together with two known analogs 3, 4 were isolated from the rhizomes of Alpinia officinarum. The new compounds were elucidated to be (5S)-5-hydroxy-7-(3, 4-dihydroxyphenyl)-1-phenyl-3-heptanone 1 and (5R)-5-hydroxy-7-(3-methoxy-4, 5-dihydroxyphenyl)-1-phenyl-3-heptanone 2 based on their spectral analysis. Compound 4 showed moderate cytotoxicity against human tumor cell lines; HepG2 and SF-268, while no significant effect were found for compounds 1–3 [58]. Ning and Pan tested compounds 1–4 for their cytotoxic activity against human cancer cell lines. They showed that compound 4 even at low concentration (6–10 μg/ml) exhibited potent cytotoxicity against all cell lines [59,60]. Galangal rhizomes also contains pinocembrin (5, 7-dihdroxyflavanone) that shows cytotoxicity against some cancer cells including normal lung fibroblasts with relative nontoxicity to human umbilical cord endothelial cells. Pinocembrin induced loss of mitochondrial membrane potential with subsequent release of cytochrome c and processing of caspase-9 and -3 colon cancer cell line HCT116 [50]. Therefore, it seems likely that potential compounds responsible for the inhibitory effect of galangal on tumor cell growth are its ingredients. Our data showed that galangal extract inhibits significantly proliferation of the human breast adenocarcinoma cells in a concentration- and time-dependent manner versus the normal human fetal lung fibroblast. The morphological features also confirmed these results. We also showed that apoptosis induced by the galangal extract was significantly higher than control, and the percentage of the both early and late apoptotic cells statistically increased by increasing galangal concentration. On the other hand, although no significant difference was detected between the percentage of early and late apoptotic cells at lower concentration, however, the number of the late apoptotic cells versus the early apoptotic cells at higher concentrations of the galangal treated cells was statically significant.

Generally, apoptosis can occur via two fundamental pathways: (1) the mitochondrial or intrinsic pathway; and, (2) the death receptor or extrinsic pathway [61]. The intrinsic pathway is triggered by release of mitochondrial proteins, such as cytochrome c, which bind with Apaf-1 and procaspase-9 in a dATP-dependent manner to form the apoptosome [62]. The apoptosome can induce activation of caspase- 9, thereby initiating apoptotic caspase cascades [63,64]. Conversely, the extrinsic pathway is initiated by the interaction of ligands with their respective death receptors, sequentially leading to cleavage of initiator caspase-8. The active caspase-8 cleaves executioner caspase-3, resulting in apoptosis [65]. Activated caspase-9 and -8 further initiate activation of the caspase cascade, leading to biochemical and morphological changes associated with apoptosis [66,67]. Thus caspases have been shown to be activated during apoptosis in many cells and play critical roles in both initiation and execution of apoptosis [68]. The intrinsic pathway of apoptosis is regulated by the Bcl-2 family of proteins. Anti-apoptotic (e.g. Bcl-2 and Bcl-xL) and pro-apoptotic (e.g. Bad, Bax and Bak) are two of the major members in Bcl-2 family [69-71]. Anti-apoptotic Bcl-2 and Bcl-xL inhibit apoptosis by sequestering proforms of capsases or by preventing the release of mitochondrial apoptogenic factors [72,73]. Bad, Bax and Bak inhibit Bcl-2 activity and promote apoptosis [74]. These experimental findings suggest that A.galangal extract induced MCf-7 cells apoptosis however, further detailed investigations of this mechanism are warranted to obtain definite conclusions.

## Conclusion

Overall, this study showed that the galangal extract may contain bioactive compounds that inhibit the proliferation of the human breast adenocarcinoma cell line (MCF-7) with the involvement of apoptosis or programmed cell death. Further research focuses on galangal gradients are needed to fully recognize the mechanisms involved in cell death. The ethanolic extract of galangal could be also considered as a promising chemotherapeutic agent in breast cancer treatment.

## Competing interests

The authors confirm that this article content has no conflict of interest.

## Authors’ contributions

SS: Supervisor, writing manuscript, design of the manuscrript. MH: Writing manuscript, Help to design the manuscript, doing MTT experiment. JTA: Writing manuscript, doing the morphological experiment. MH: Doing Anniexin /PI, Flocytometry experimetand and also analytical analysis. All authors read and approved the final manuscript.

## Pre-publication history

The pre-publication history for this paper can be accessed here:

http://www.biomedcentral.com/1472-6882/14/192/prepub
